# Supplementary figures and images for: Circadian oscillations in Trichoderma atroviride and the role of core clock components in secondary metabolism, development, and mycoparasitism against the phytopathogen Botrytis cinerea
Source: eLife. 2022 Aug 11;11:e71358. doi: 10.7554/eLife.71358 (PMC9427114; doi:10.7554/eLife.71358)

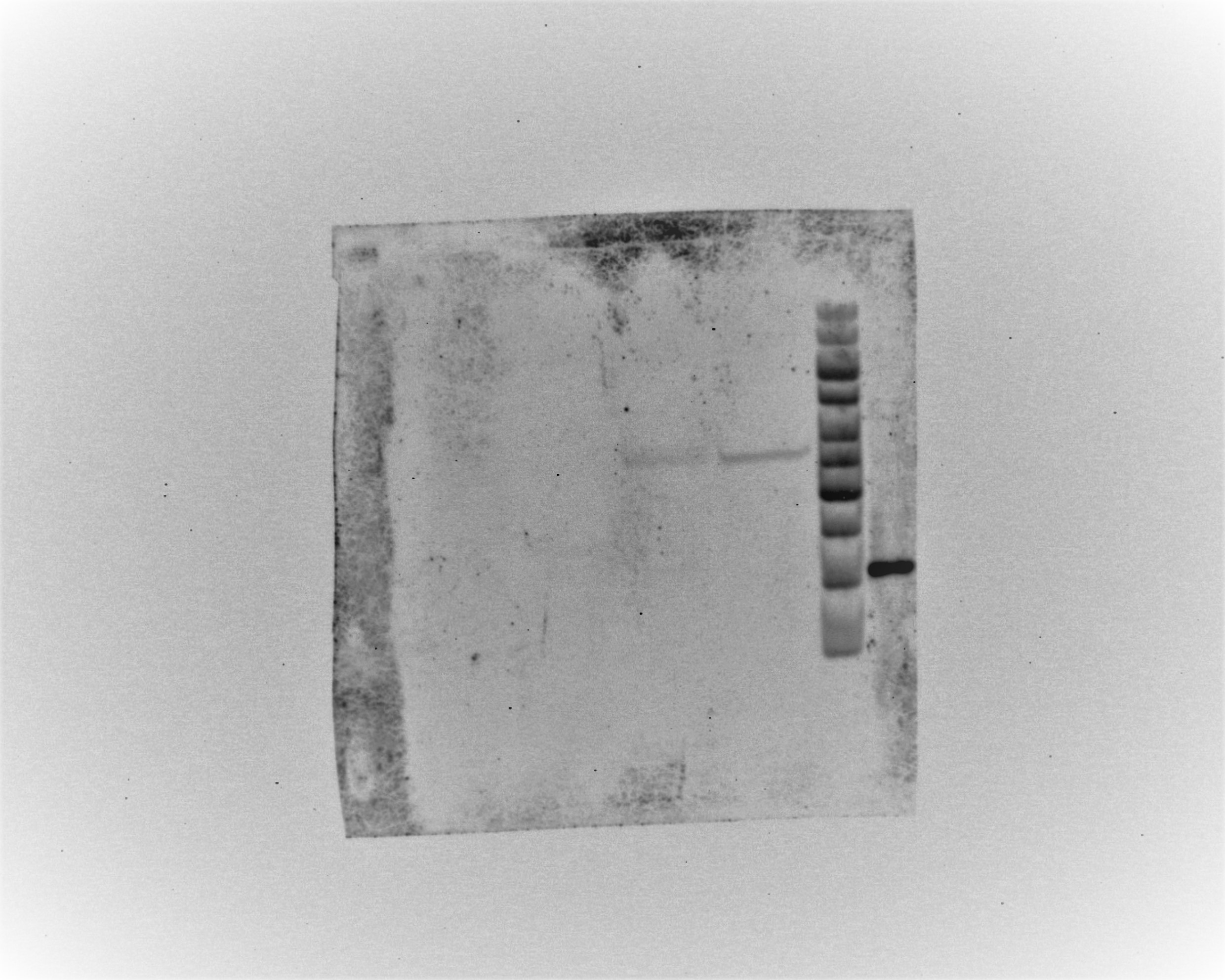

Supplement: Figure 1—figure supplement 1—source data 1. — Original Southern blot of TaFRQLUC (strains 2 and 4) and TaWT. [file elife-71358-fig1-figsupp1-data1.jpg]

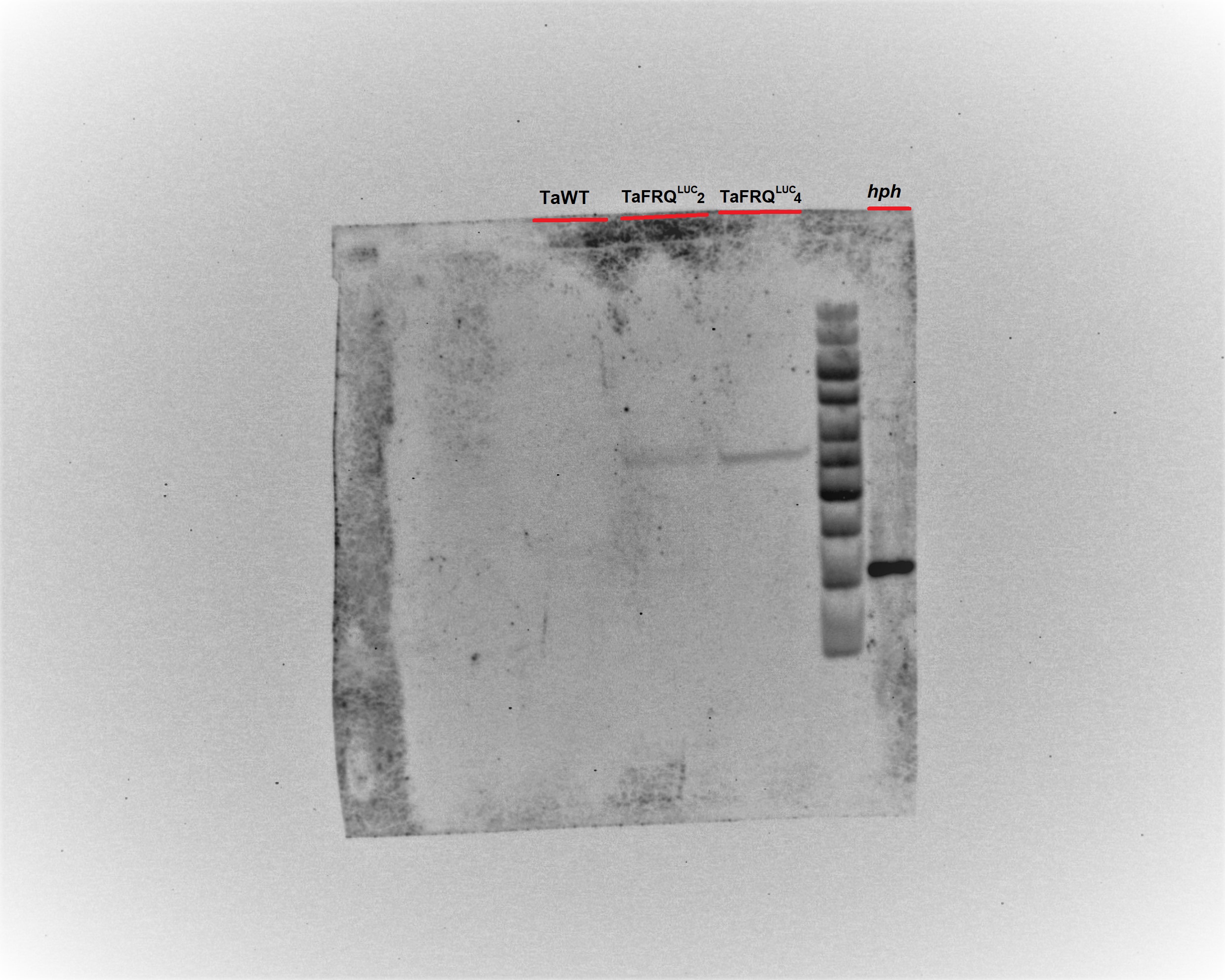

Supplement: Figure 1—figure supplement 1—source data 2. — Labeled Southern blot of TaFRQLUC (strains 2 and 4) and TaWT. [file elife-71358-fig1-figsupp1-data2.jpg]

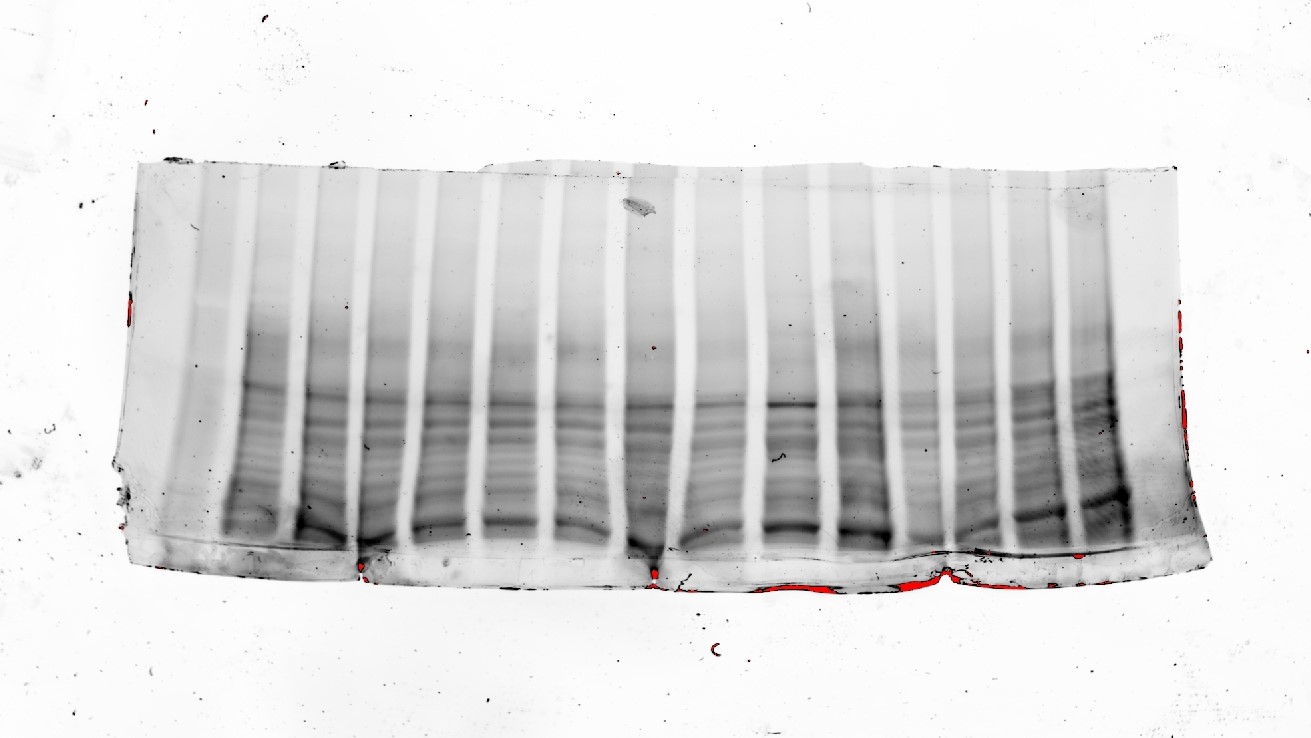

Supplement: Figure 1—figure supplement 5—source data 1. — Original protein gel of TaFRQLUC time course. [file elife-71358-fig1-figsupp5-data1.jpg]

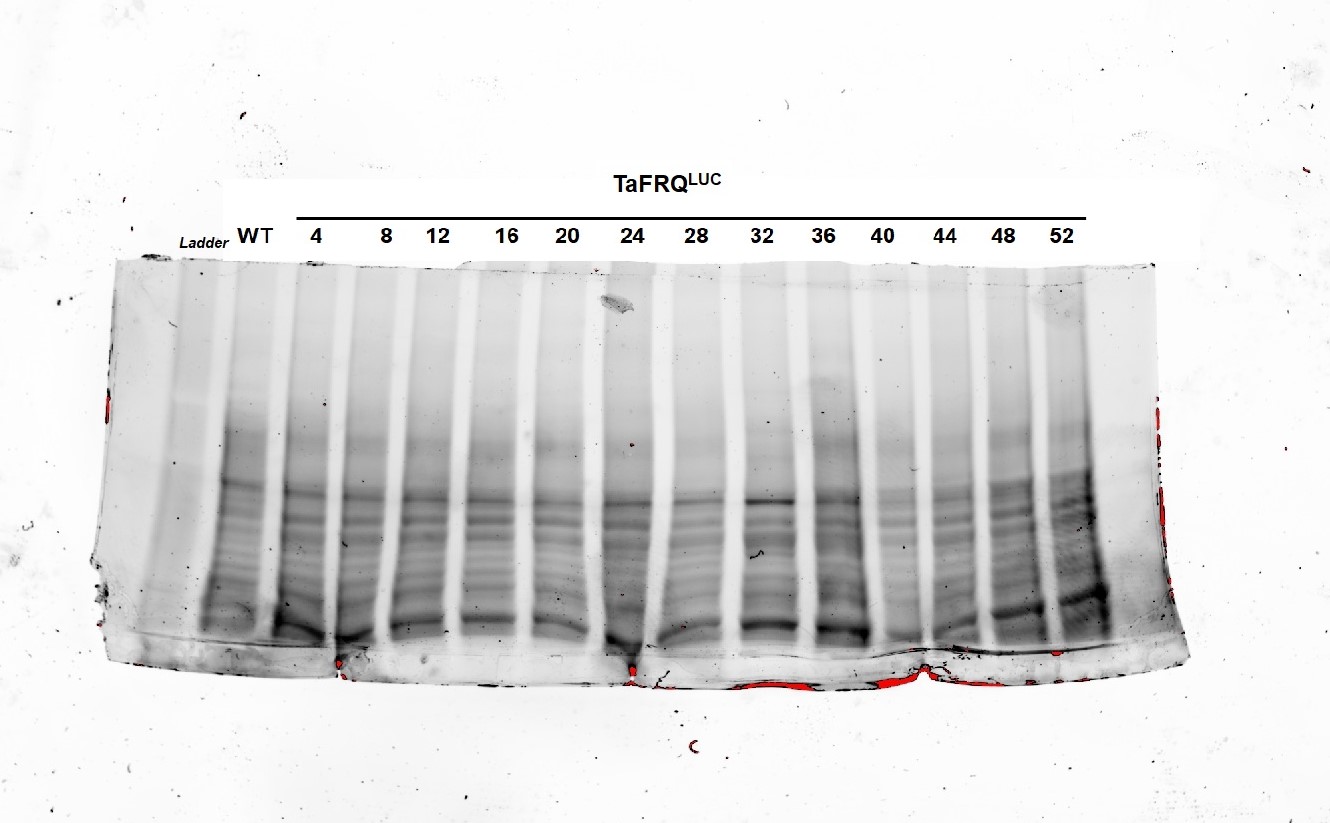

Supplement: Figure 1—figure supplement 5—source data 2. — Labeled protein gel of TaFRQLUC time course. [file elife-71358-fig1-figsupp5-data2.jpg]

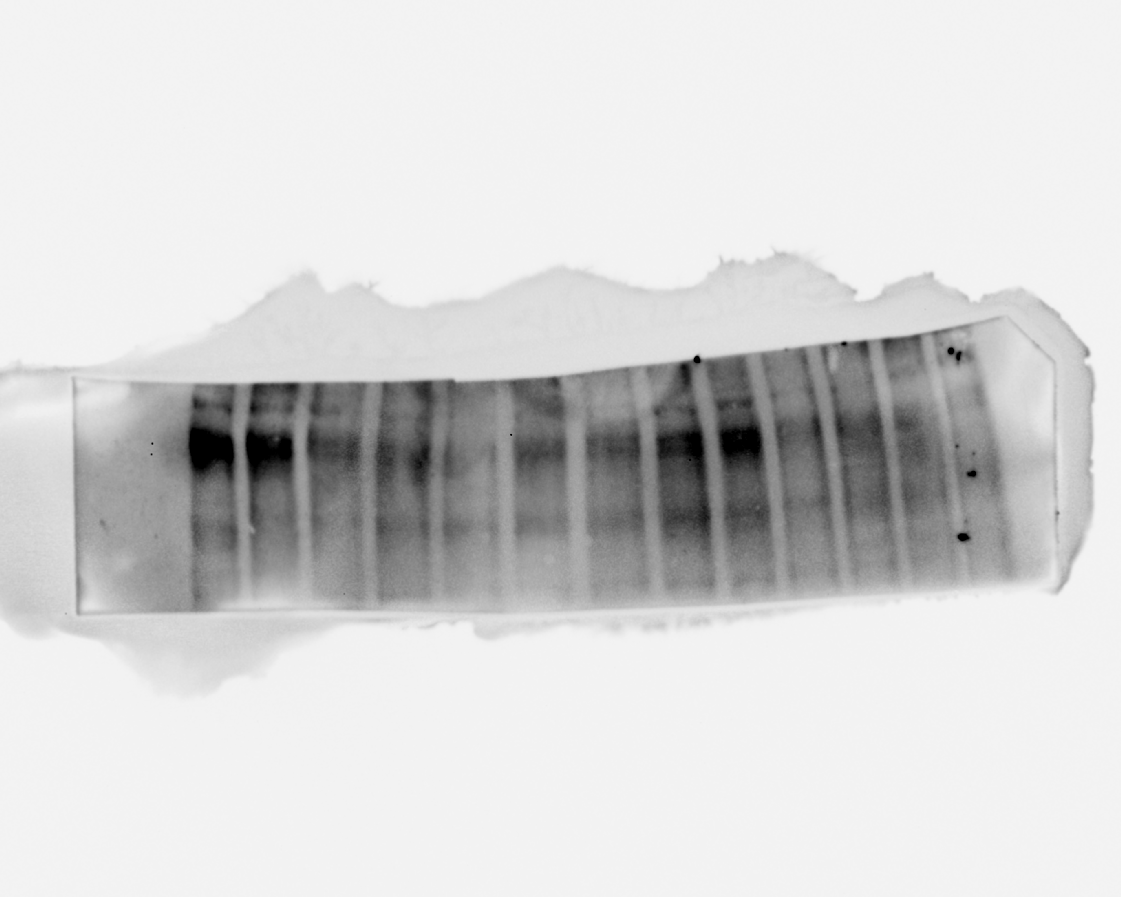

Supplement: Figure 1—figure supplement 5—source data 3. — Original Western blot of TaFRQLUC time course. [file elife-71358-fig1-figsupp5-data3.tif]

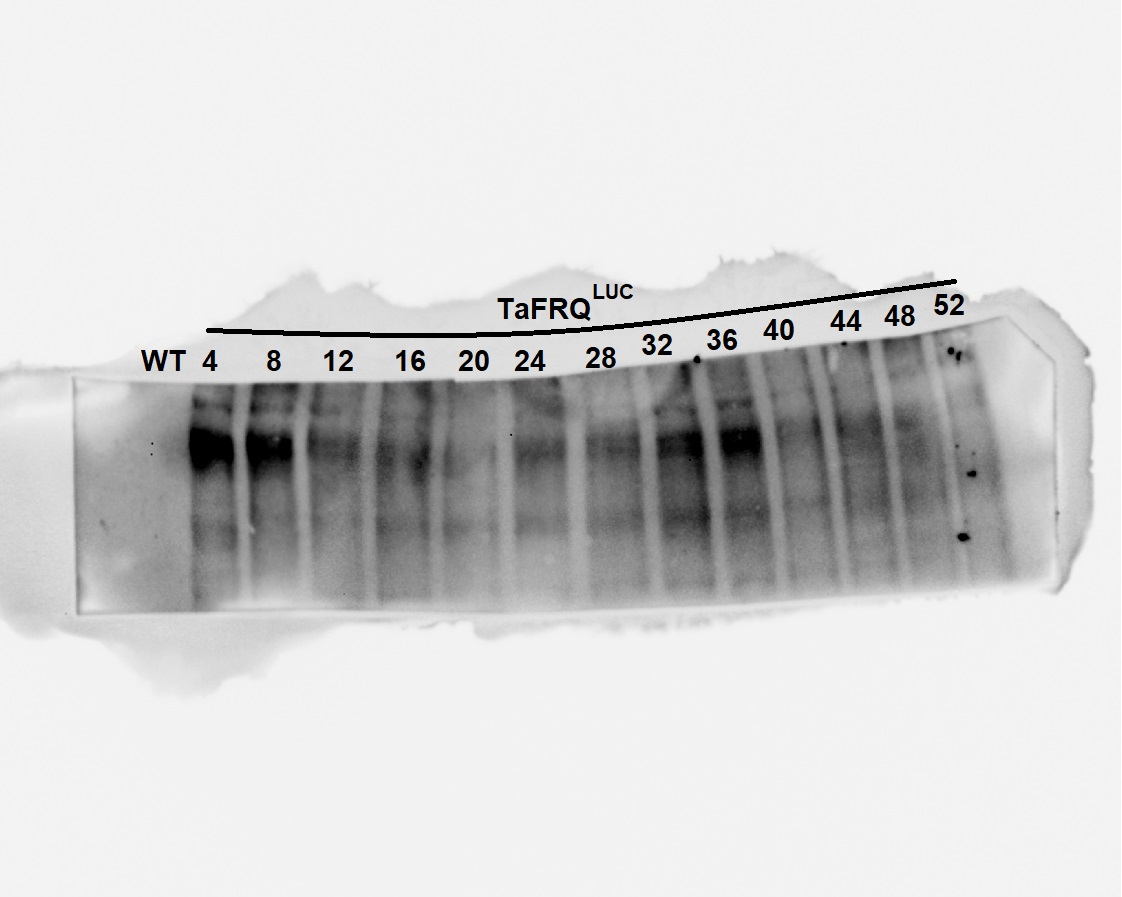

Supplement: Figure 1—figure supplement 5—source data 4. — Labeled Western blot of TaFRQLUC time course. [file elife-71358-fig1-figsupp5-data4.jpg]

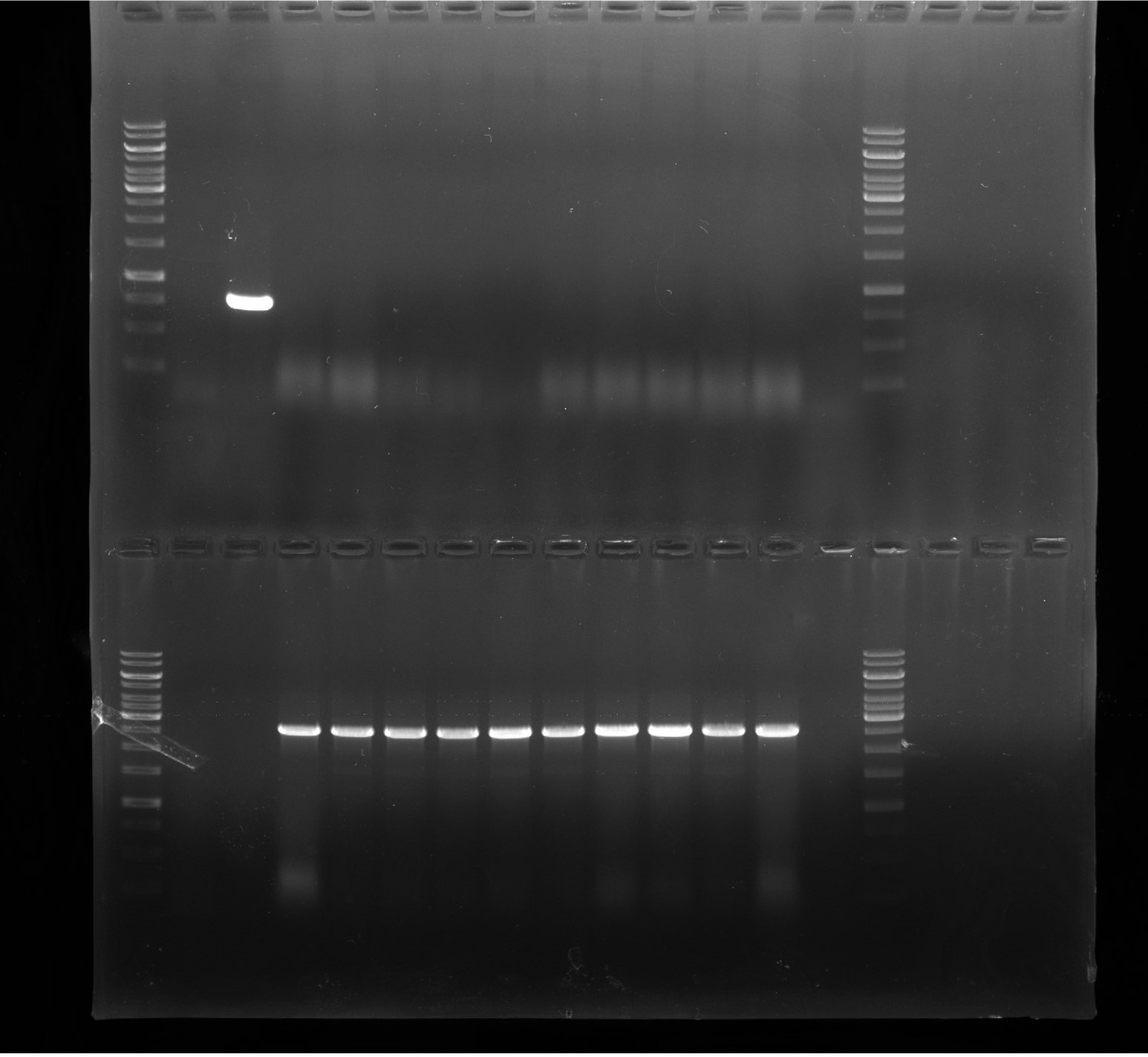

Supplement: Figure 2—figure supplement 2—source data 1. — Original agarose gel of Δncfrq::tafrq diagnostic PCRs. [file elife-71358-fig2-figsupp2-data1.jpg]

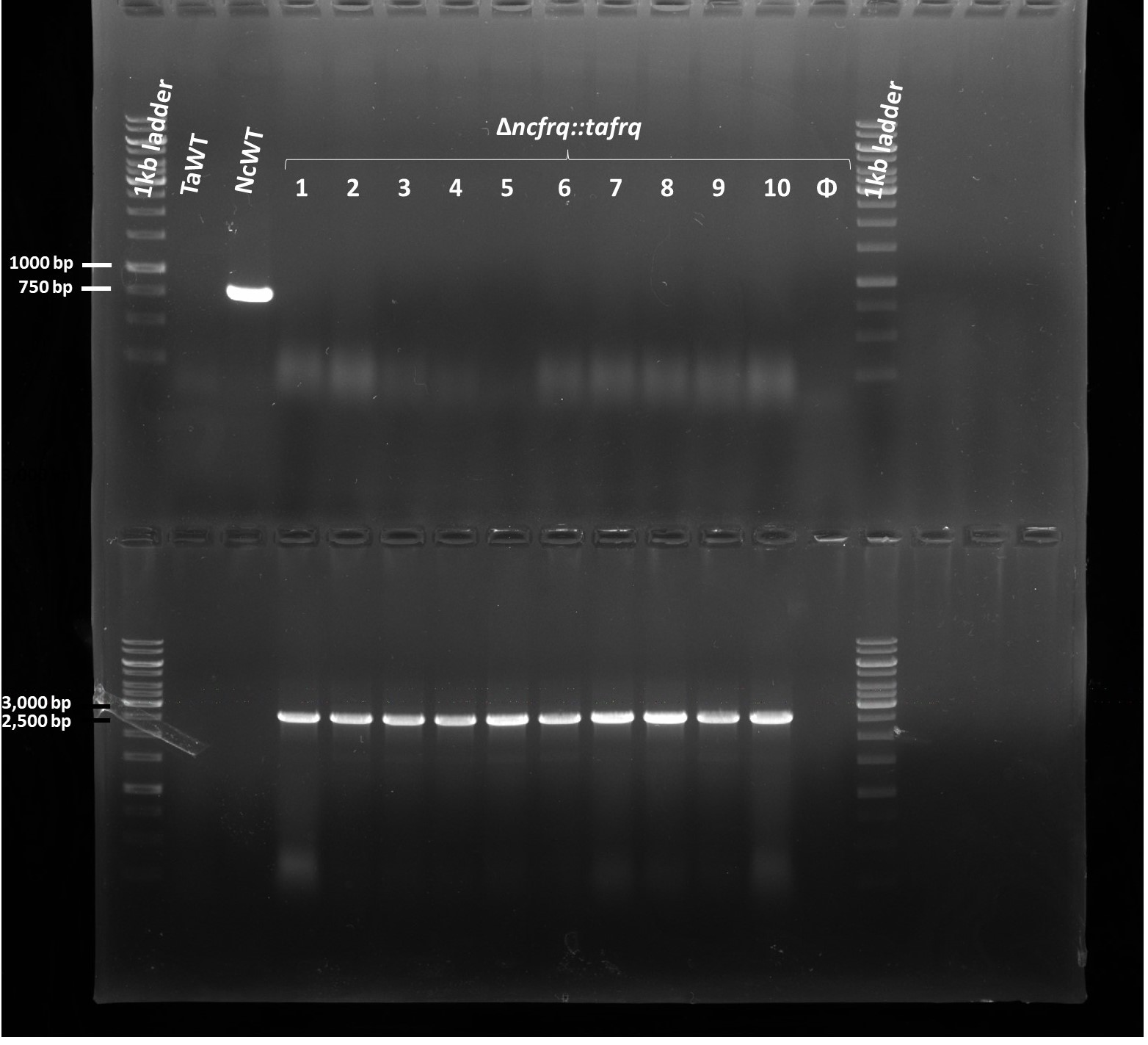

Supplement: Figure 2—figure supplement 2—source data 2. — Labeled agarose gel of Δncfrq::tafrq diagnostic PCRs. [file elife-71358-fig2-figsupp2-data2.jpg]

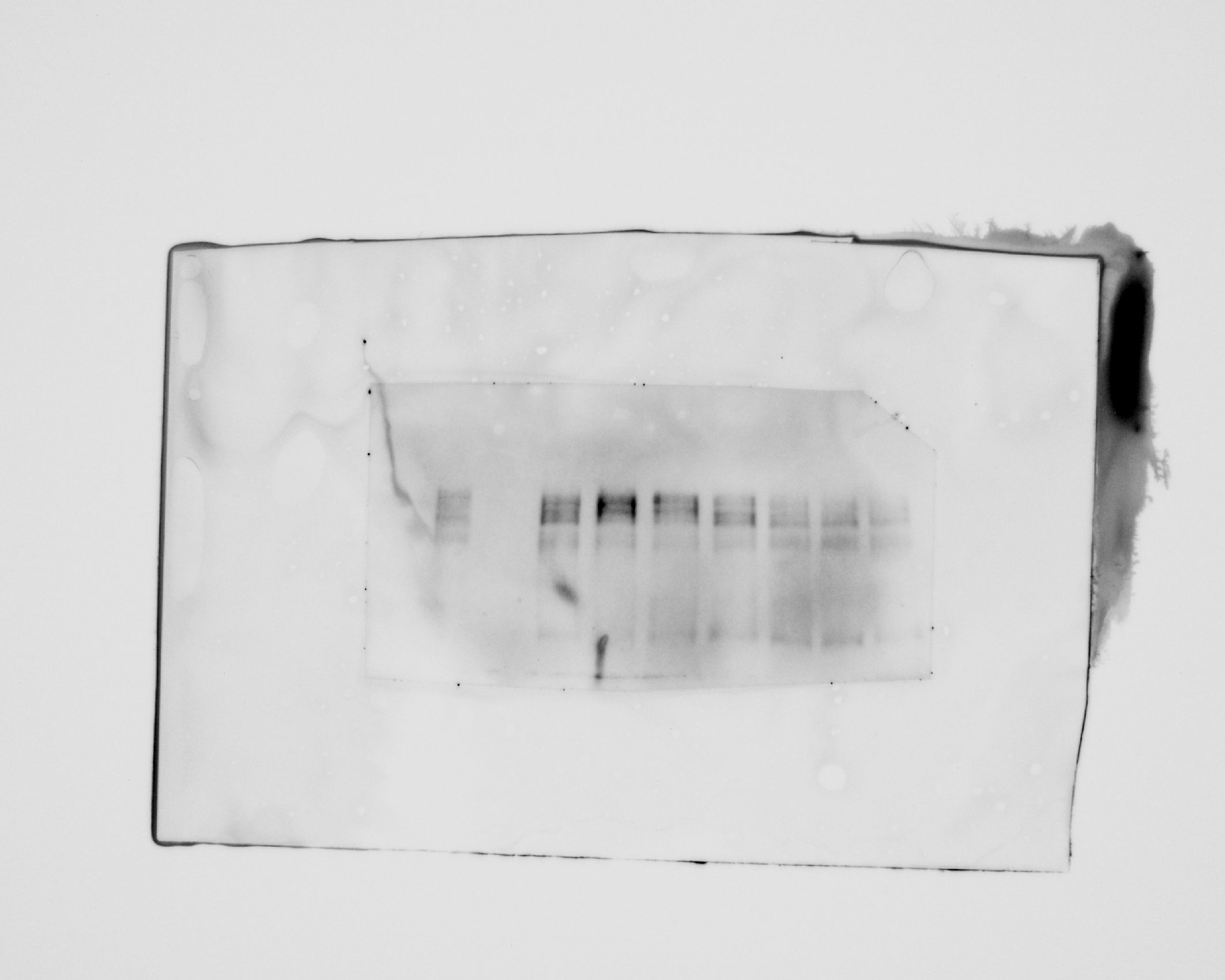

Supplement: Figure 2—figure supplement 2—source data 3. — Original Δncfrq::tafrq Western blot anti-TaFRQ-V5 time course. [file elife-71358-fig2-figsupp2-data3.tif]

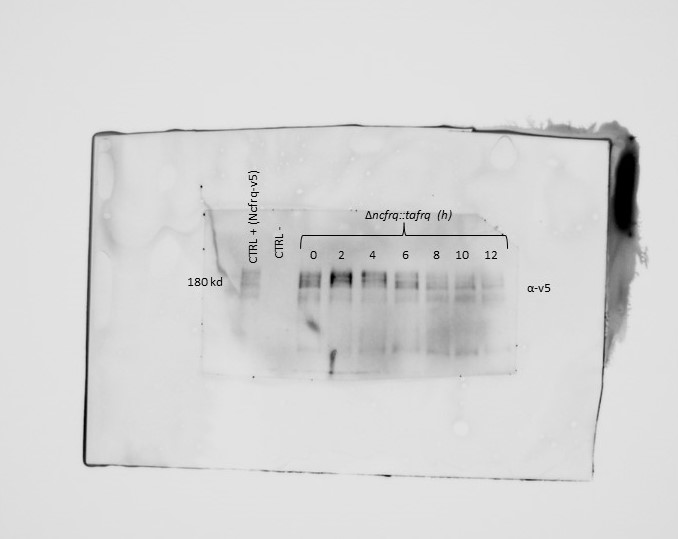

Supplement: Figure 2—figure supplement 2—source data 4. — Labeled Δncfrq::tafrq Western blot anti-TaFRQ-V5 time course. [file elife-71358-fig2-figsupp2-data4.jpg]

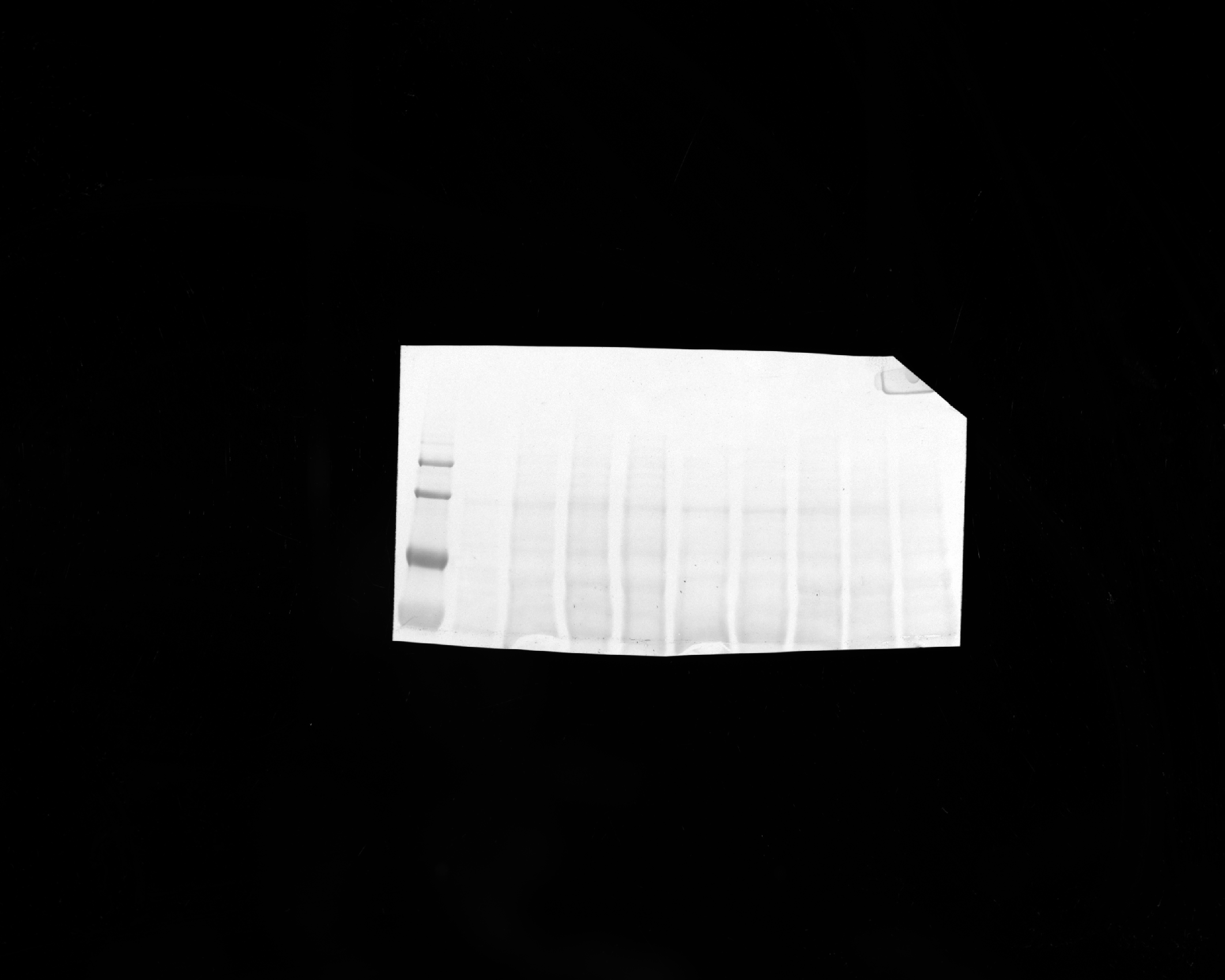

Supplement: Figure 2—figure supplement 2—source data 5. — Original Δncfrq::tafrq stained gel TaFRQ-V5 time course. [file elife-71358-fig2-figsupp2-data5.tif]

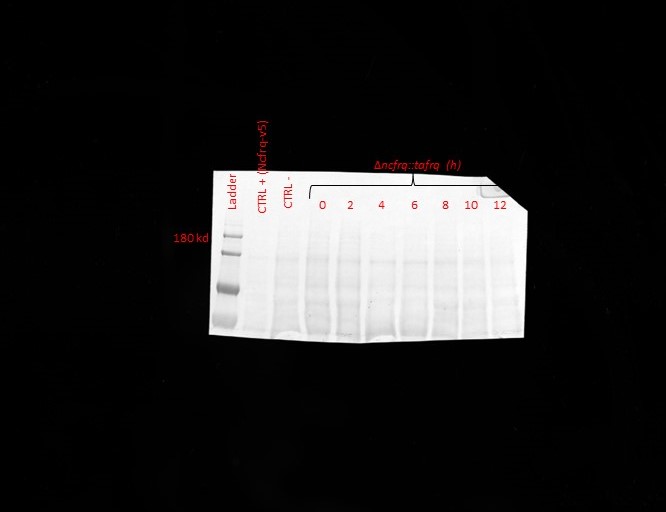

Supplement: Figure 2—figure supplement 2—source data 6. — Labelled Δncfrq::tafrq stained gel TaFRQ-V5 time course. [file elife-71358-fig2-figsupp2-data6.jpg]

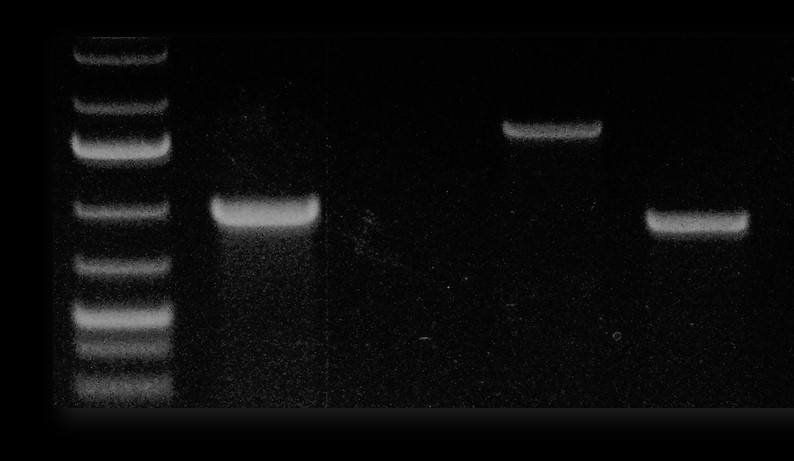

Supplement: Figure 3—figure supplement 1—source data 1. — Original gel of Δtafrq diagnostic PCRs. [file elife-71358-fig3-figsupp1-data1.jpg]

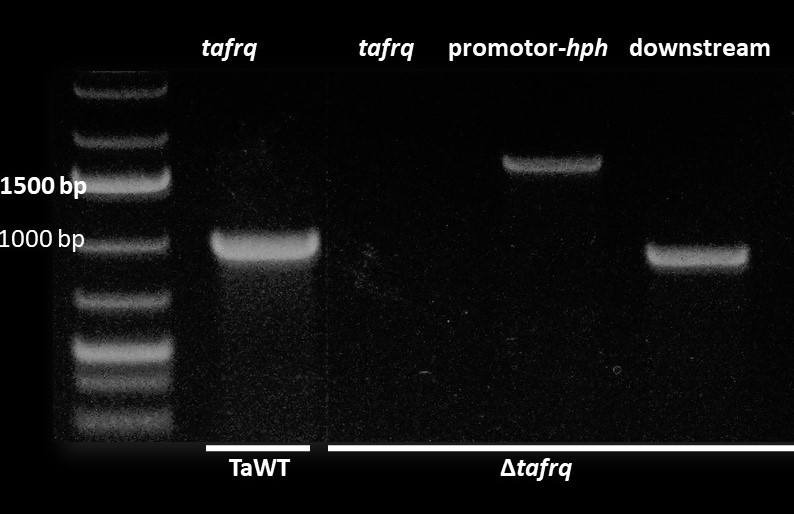

Supplement: Figure 3—figure supplement 1—source data 2. — Labeled gel of Δtafrq diagnostic PCRs. [file elife-71358-fig3-figsupp1-data2.jpg]

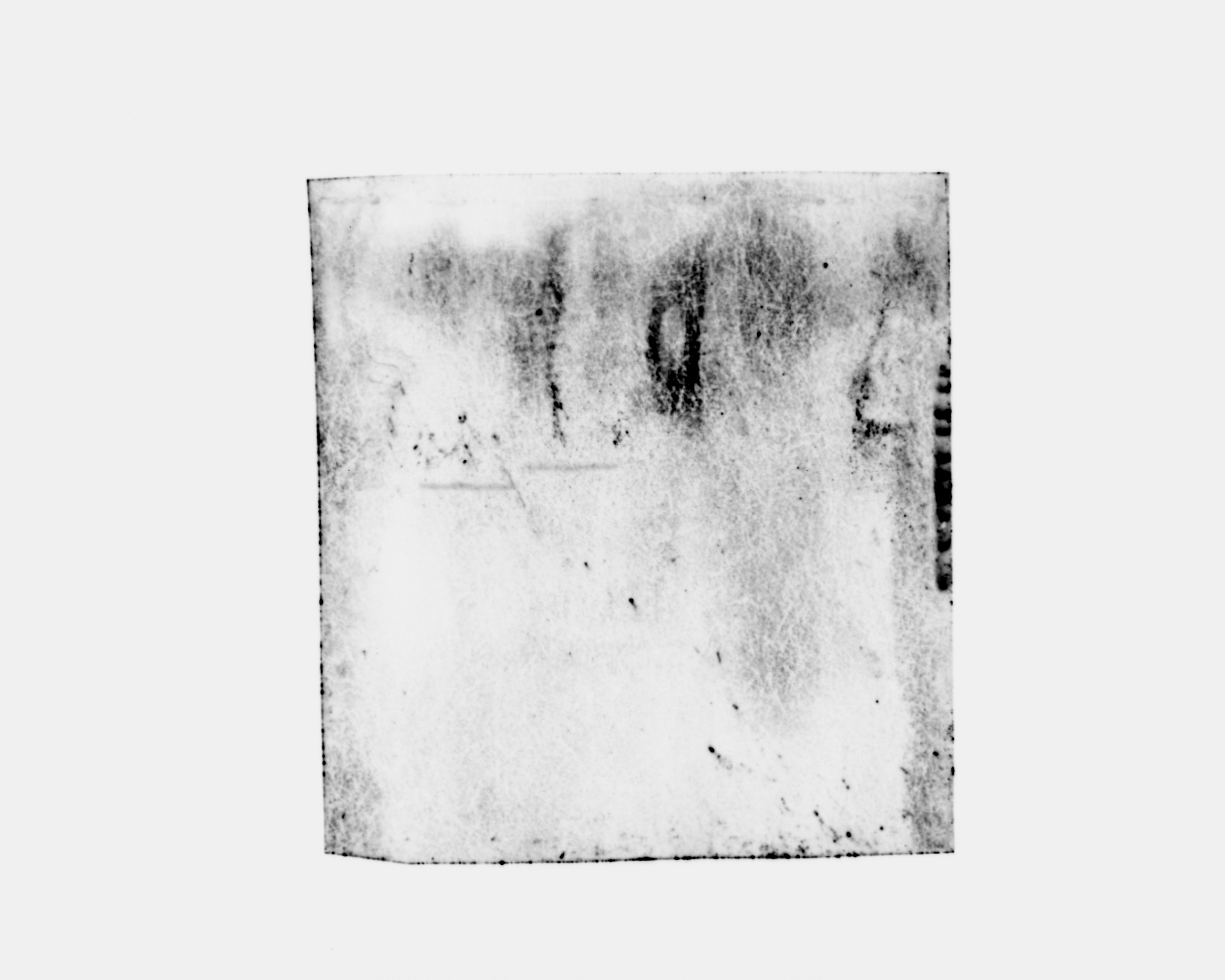

Supplement: Figure 3—figure supplement 1—source data 3. — Original Southern blot of Δtafrq and TaWT. [file elife-71358-fig3-figsupp1-data3.jpg]

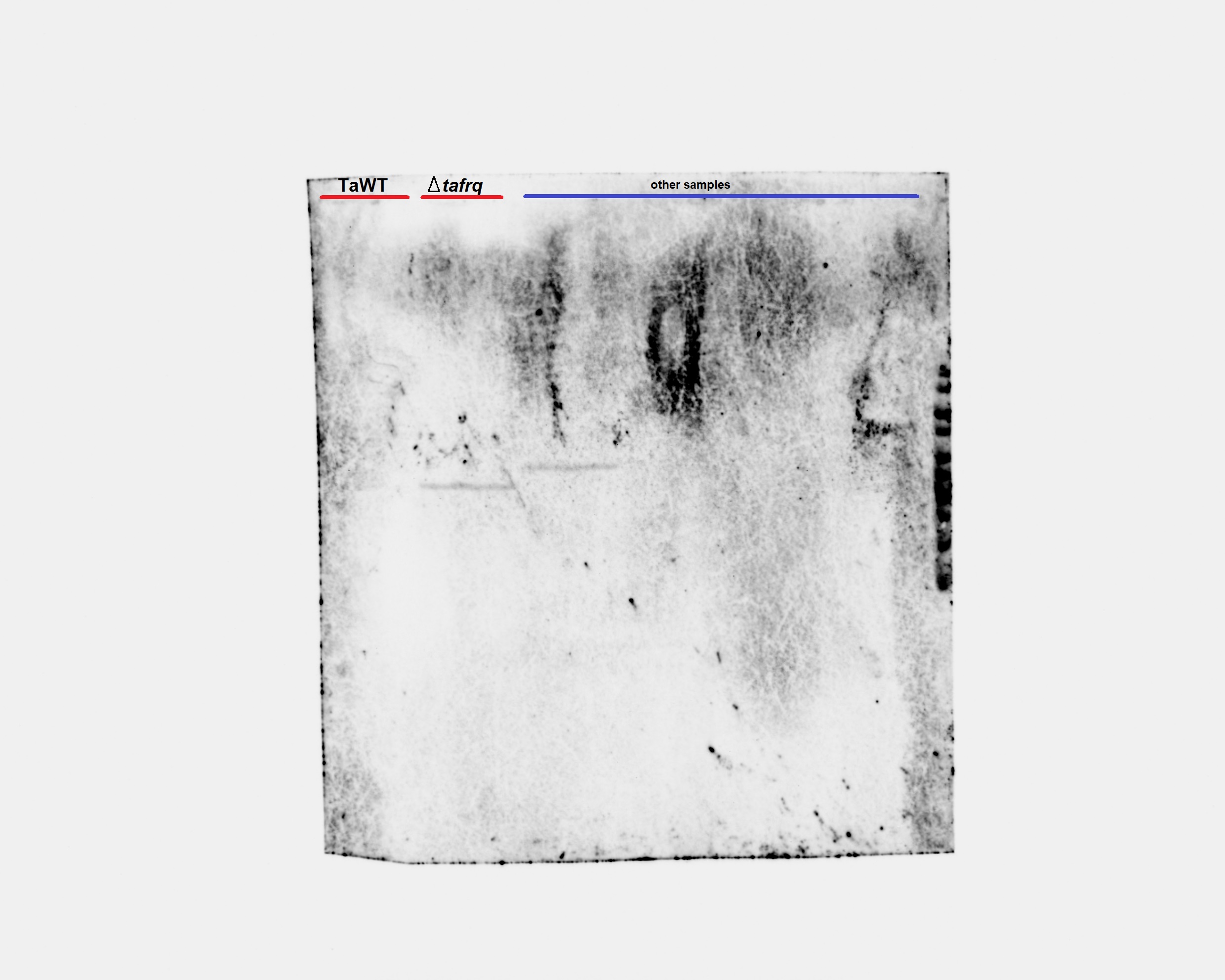

Supplement: Figure 3—figure supplement 1—source data 4. — Labeled Southern blot of Δtafrq and TaWT. [file elife-71358-fig3-figsupp1-data4.jpg]

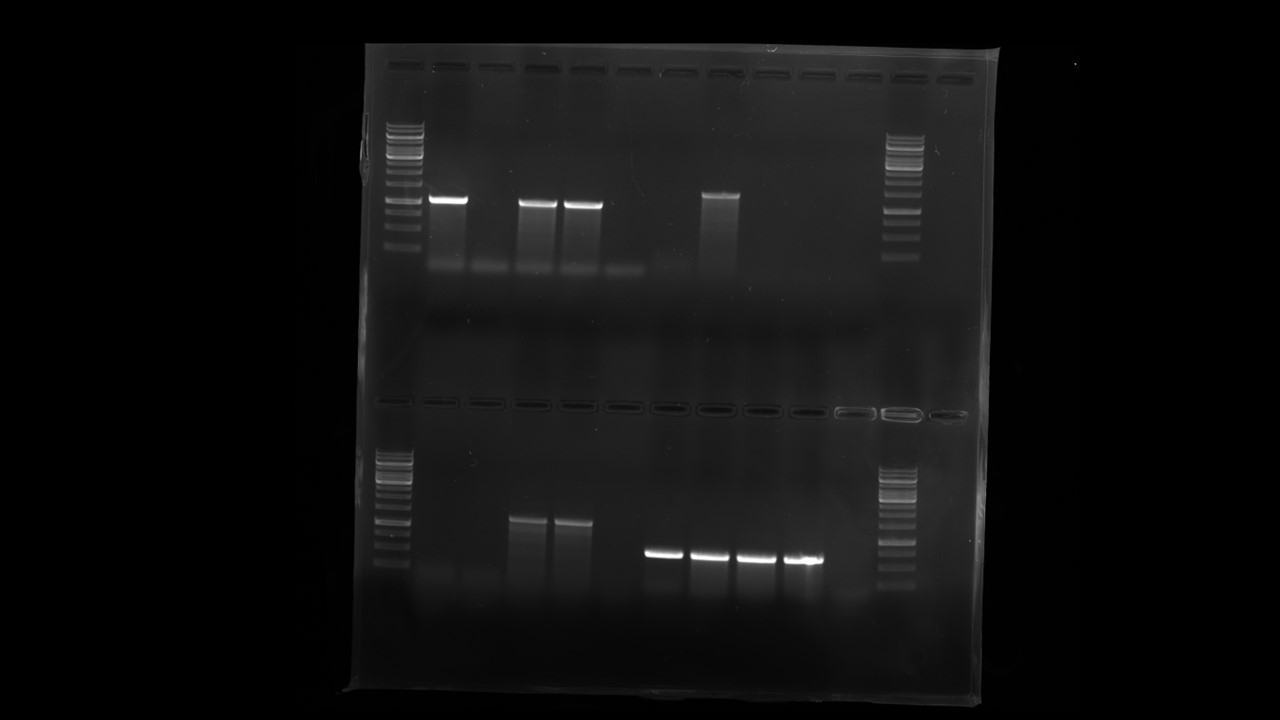

Supplement: Figure 3—figure supplement 3—source data 1. — Original agarose gel of ∆ncfrq::tafrq diagnostic PCRs. [file elife-71358-fig3-figsupp3-data1.jpg]

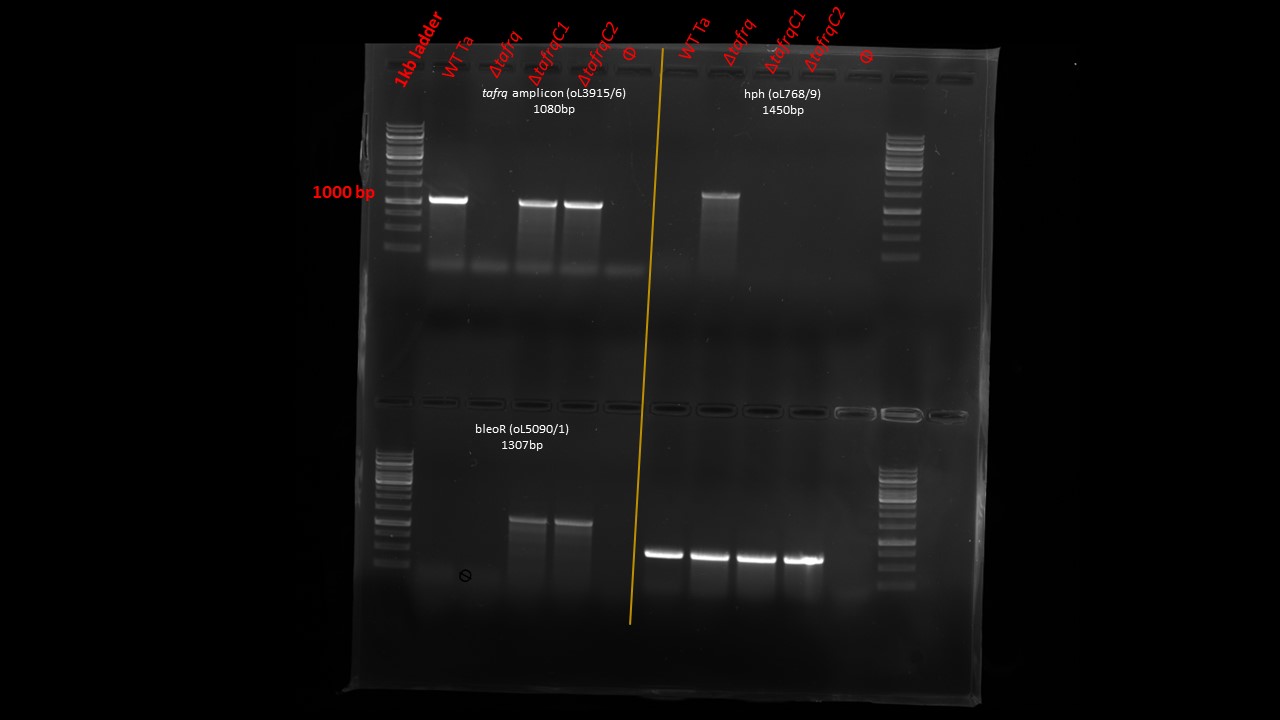

Supplement: Figure 3—figure supplement 3—source data 2. — Labeled agarose gel of ∆ncfrq::tafrq diagnostic PCRs. [file elife-71358-fig3-figsupp3-data2.jpg]

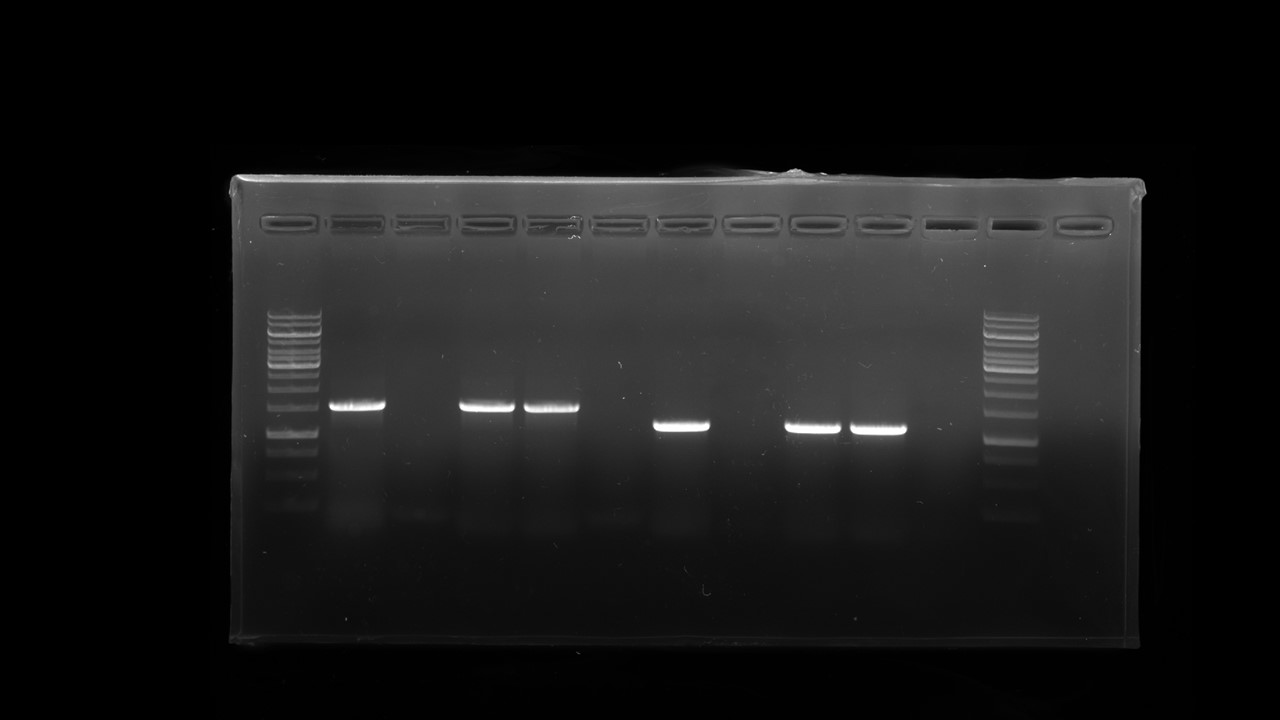

Supplement: Figure 3—figure supplement 3—source data 3. — Original agarose gel of ∆ncfrq::tafrq diagnostic PCRs. [file elife-71358-fig3-figsupp3-data3.jpg]

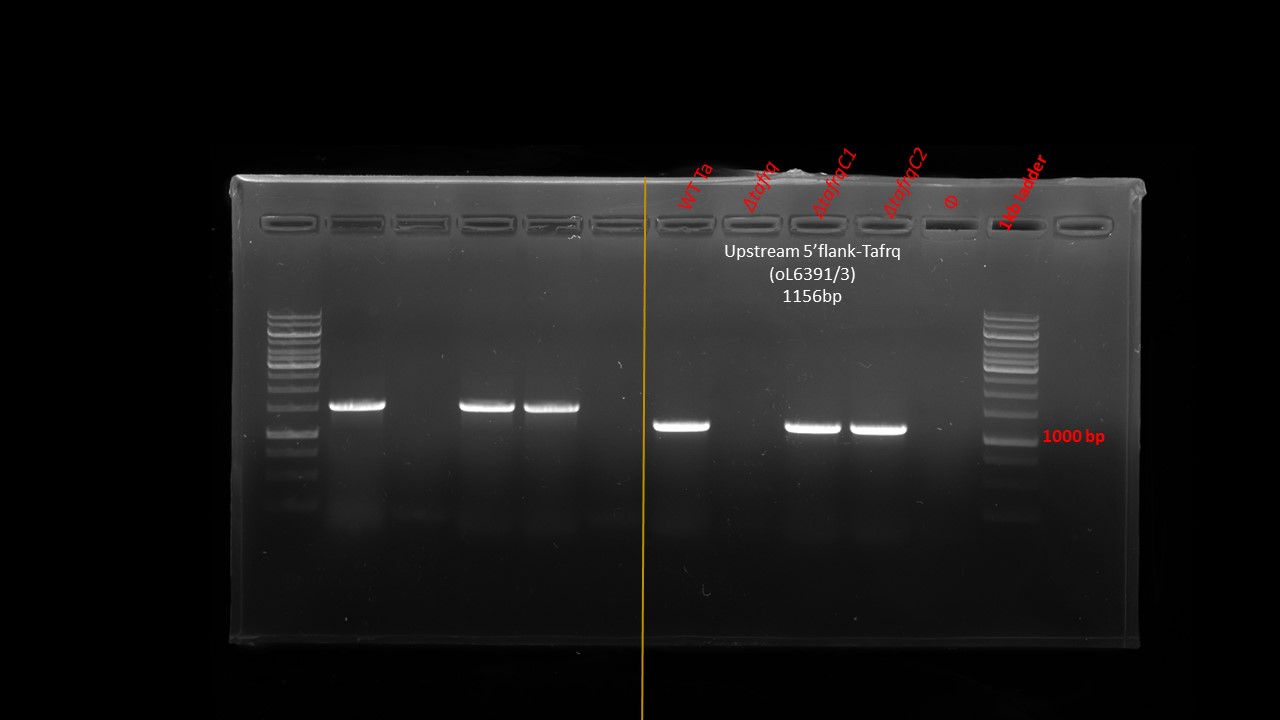

Supplement: Figure 3—figure supplement 3—source data 4. — Labeled agarose gel of ∆ncfrq::tafrq diagnostic PCRs. [file elife-71358-fig3-figsupp3-data4.jpg]
