# Supplementary material for: Circadian oscillations in Trichoderma atroviride and the role of core clock components in secondary metabolism, development, and mycoparasitism against the phytopathogen Botrytis cinerea
Source: eLife. 2022 Aug 11;11:e71358. doi: 10.7554/eLife.71358 (PMC9427114; doi:10.7554/eLife.71358)
Supplement: Supplementary file 1. [file elife-71358-supp1.docx]

**Table S1.** Culture media used for *in vivo* luminescence assessment.

| **Culture media** | **Composition** |
| --- | --- |
| **PDA** | Potato Dextrose Agar (Difco). 39 grs per liter. |
| **PDB** | Potato Dextrose Broth (Difco). 12 grs per liter. |
| **Gamborg B5 2% glucose** | 2% glucose, 0.31625% Gamborg B5 (Duchefa Biochemie), 1.5% agar. |
| **MMV 1% Glucose** | 1% Vogel’s salts, 1% glucose, 1.5% agar. |
| **LNN 0.03% Glucose** | 1% Vogel’s salts, 0.03% glucose, 0.05% arginine, 50ug biotin,1.5% agar. |
| **LNN 0.3% Glucose** | 1% Vogel’s salts, 0.3% glucose, 0.05% arginine, 50ug biotin,1.5% agar. |
| **Maltose** | 1% Vogel’s salts, 0.5% maltose, 1.5% agar. |
| **MMV 1% Sucrose** | 1% Vogel’s salts, 1% sucrose, 1.5% agar. |
| **GYEC** | 0.5% glucose, 0.3% yeast extract (Difco), 0.5% casaminoacids (Difco), 1.5% agar. |
| **GYEC+peas** | 0.5% glucose, 0.3% yeast extract (Difco), 0.5% casaminoacids (Difco), 0.5% ground peas, 1.5% agar. |
