# Supplementary material for: Circadian oscillations in Trichoderma atroviride and the role of core clock components in secondary metabolism, development, and mycoparasitism against the phytopathogen Botrytis cinerea
Source: eLife. 2022 Aug 11;11:e71358. doi: 10.7554/eLife.71358 (PMC9427114; doi:10.7554/eLife.71358)
Supplement: Supplementary file 2. [file elife-71358-supp2.docx]

**Table S2.** Period analysis of transcriptional reporters in *T. atroviride* using BioDare platform.

| **Data** | **Period** | **P.Std** | **Phase** | **Ph.Std** | **Amp.** | **Amp.Std** |
| --- | --- | --- | --- | --- | --- | --- |
| ***_Nc_c-box-luc*** | 25.67 | 1.01 | 3.53 | 1.96 | 4.86e+0 | 1.92e+0 |
| ***ccg-9_prom_-luc*** | NaN | NaN | NaN | NaN | NaN | NaN |
| ***sod_prom_-luc*** | NaN | NaN | NaN | NaN | NaN | NaN |
| ***con-10_prom_-luc*** | NaN | NaN | NaN | NaN | NaN | NaN |
| ***gpdh_prom_-luc*** | NaN | NaN | NaN | NaN | NaN | NaN |
