# Supplementary material for: Circadian oscillations in Trichoderma atroviride and the role of core clock components in secondary metabolism, development, and mycoparasitism against the phytopathogen Botrytis cinerea
Source: eLife. 2022 Aug 11;11:e71358. doi: 10.7554/eLife.71358 (PMC9427114; doi:10.7554/eLife.71358)
Supplement: Supplementary file 3. [file elife-71358-supp3.docx]

**Table S3.** Mean overgrowth area of *T. atroviride* over *B. cinerea* in all confrontation assays performed.

|  | **Condición** | **B05.10** | ***Δbcwcl1*** | ***Δbcfrq1*** |
| --- | --- | --- | --- | --- |
| **TaWT** | LL | 19.29 ± 3.58 | 73.24± 14.03 | 23.55± 9.85 |
|  | DD | 57.46 ±18.06 | 85.75± 8.06 | 59.76± 4.07 |
|  | LD AM | 69.35± 2.06 | 85.09± 5.83 | 48.54± 9.69 |
|  | DL PM | 37.65± 7.32 | 23.02± 7.29 | 49.21± 5.13 |
| ***Δblr1*** | LL | 34.68± 17.38 | 33.04± 7.06 | 41.91± 12.52 |
|  | DD | 89.86± 2.49 | 90.41± 3.57 | 79.35± 9.29 |
|  | LD AM | 83.46± 5.95 | 92.25± 3.07 | 82.36± 7.20 |
|  | DL PM | 72.86± 6.42 | 82.26± 15.48 | 63.85± 2.89 |
| ***Δtafrq*** | LL | 7.32± 12.68 | 18.54± 6.93 | 0.00 |
|  | DD | 50.27± 15.21 | 80.30± 1.48 | 62.25± 8.77 |
|  | LD AM | 73.07± 8.63 | 83.83± 7.49 | 58.94± 7.88 |
|  | DL PM | 32.76± 7.41 | 31.18± 6.19 | 53.88± 6.62 |
