# Supplementary material for: Circadian oscillations in Trichoderma atroviride and the role of core clock components in secondary metabolism, development, and mycoparasitism against the phytopathogen Botrytis cinerea
Source: eLife. 2022 Aug 11;11:e71358. doi: 10.7554/eLife.71358 (PMC9427114; doi:10.7554/eLife.71358)
Supplement: Supplementary file 4. — The period was estimated using FFT-NLSS, and rhythmicity was evaluated using BD2eJTK in the BioDare platform. [file elife-71358-supp4.docx]

**Table S4.** Circadian metabolites of *T. atroviride.* The period was estimated using FFT NLSS, and rhythmicity was evaluated using BD2eJTK in the BioDare platform.

| **TaWT** | | | | | | |
| --- | --- | --- | --- | --- | --- | --- |
| **Group Label** | N | Period | Period Std | GOF | ERR | Rhythmic |
| **63.83*** | 4 | 24.60 | 0.10 | 0.76 | 0.91 | **TRUE** |
| **73.89** | 5 | 25.14 | 0.46 | 0.64 | 0.69 | **TRUE** |
| **81.59** | 4 | 24.44 | 0.11 | 0.74 | 0.87 | **TRUE** |
| **90.83*** | 6 | 25.12 | 0.28 | 0.63 | 0.83 | **TRUE** |
| **95.78** | 5 | 24.34 | 0.50 | 0.54 | 0.58 | **TRUE** |
| **148.97** | 5 | 24.72 | 0.59 | 0.68 | 0.78 | **TRUE** |
| **163** | 4 | 24.53 | 0.32 | 0.63 | 0.8 | **TRUE** |
| **179.96** | 4 | 24.62 | 0.41 | 0.68 | 0.78 | **TRUE** |
| **181.01** | 4 | 24.60 | 0.38 | 0.74 | 0.77 | **TRUE** |
| **195.03** | 4 | 23.48 | 0.46 | 0.63 | 0.8 | **TRUE** |
| **211.98** | 4 | 24.07 | 0.74 | 0.7 | 0.79 | **TRUE** |
| **388.87*** | 3 | 24.00 | NA | NA | NA | **TRUE** |
| **389.77*** | 4 | 24.00 | NA | NA | NA | **TRUE** |
| **461.89** | 4 | 24.16 | 0.22 | 0.48 | 0.63 | **TRUE** |
| **462.89** | 4 | 24.21 | 0.19 | 0.62 | 0.76 | **TRUE** |
| **463.88** | 4 | 24.08 | 0.28 | 0.49 | 0.64 | **TRUE** |
| **464.92** | 4 | 24.31 | 0.30 | 0.45 | 0.66 | **TRUE** |
| ***Δtafrq*** | | | | | | |
| **Group Label** | N | Period | Period Std | GOF | ERR | Rhythmic |
| **63.83*** | 3 | 24.40 | 0.15 | 0.55 | 0.95 | **TRUE** |
| **73.89*** | 1 | 23.13 | 0 | 0.45 | 0.63 | FALSE |
| **81.59*** | 2 | 24.28 | 0.06 | 0.54 | 0.93 | FALSE |
| **90.83*** | 3 | 24.70 | 1.83 | 0.81 | 0.95 | **TRUE** |
| **95.78** | 0 | NA | NA | NA | NA | FALSE |
| **148.97** | 0 | NA | NA | NA | NA | FALSE |
| **163** | 0 | NA | NA | NA | NA | FALSE |
| **179.96** | 0 | NA | NA | NA | NA | FALSE |
| **181.01*** | 1 | 24.37 | 0 | 0.55 | 0.99 | FALSE |
| **195.03** | 0 | NA | NA | NA | NA | FALSE |
| **388.87*** | 4 | 24.00 | NA | NA | NA | **TRUE** |
| **389.77*** | 4 | 24.00 | NA | NA | NA | **TRUE** |
| **211.98** | 0 | NA | NA | NA | NA | FALSE |
| **461.89** | 0 | NA | NA | NA | NA | FALSE |
| **462.89** | 0 | NA | NA | NA | NA | FALSE |
| **463.88** | 0 | NA | NA | NA | NA | FALSE |
| **464.92** | 0 | NA | NA | NA | NA | FALSE |

*For those molecules in *Δtafrq* that showed a period and/or rhythmicity according to BD2eJTK, we compared the graphics of those molecules in the mutant and TaWT strain.
