# Supplementary material for: Circadian oscillations in Trichoderma atroviride and the role of core clock components in secondary metabolism, development, and mycoparasitism against the phytopathogen Botrytis cinerea
Source: eLife. 2022 Aug 11;11:e71358. doi: 10.7554/eLife.71358 (PMC9427114; doi:10.7554/eLife.71358)
Supplement: Supplementary file 5. — Fw: direct orientation; Rv: reverse orientation. [file elife-71358-supp5.docx]

**Table S5.** List of primer used for *tafrq* replacement cassette, complementation cassette and diagnostics PCR.

 (Fw: direct orientation; Rv: reverse orientation).

| Target DNA | Primer Name | Orientation | Sequence 5’- 3’ | Size (bp) |
| --- | --- | --- | --- | --- |
| 5’ flank upstream *tafrq* gene | oL3867  oL3884 | Fw  Rv | AGCGGATAACAATTTCACACAGGAAACAGCACTTCCACCAGCCCACTCCT  AAAAATGCTCCTTCAATATCATCTTCTGTCAAATGCCTACCTAGGCTTGC | 600 |
| 3’ flank downstream *tafrq* gene | oL3807  oL3808 | Fw  Rv | GACCGGGATCCACTTAACGTTACTGAAATCAATTAGGGATTCCCTCCCCCC  GTAACGCCAGGGTTTTCCCAGTCACGACGGAAATGCGTCGAGTGCCTGT | 527 |
| *hph* | oL768  oL769 | Fw  Rv | GACAGAAGATGATATTGAAGGAGC  GATTTCAGTAACGTTAAGTGGAT | 1435 |
| Construct amplification | oL83  oL84 | Fw  Rv | GGCAGTGAGCGCAACGCAAT  ATTCAGGCTGCGCAACTGTT | 2562 |
| Upstream integration | oL1269  oL3908 | Fw  Rv | GCACCAAGCAGCAGATGATA  AAACAAAAAAAAAACAGGGCCAGTATGGTACAGTGTATACGCGCATACAG | 1680 |
| Downstream integration | oL32  oL3804 | Fw  Rv | ATGGCTGTGTAGAAGTACTC  GTAACGCCAGGGTTTTCCCAGTCACGACGAAAGTGAGACGAGATAAAGGG | 909 |
| *tafrq* ORF | oL3915  oL3916 | Fw  Rv | GAAAATTTGCAGGTCGGCATGCCCGTCGGAACCAGGCTAGCCAAACGGCA  ATAACAAATACGTCGTAGGGG | 1080 |
| 5’ flank upstream *tafrq* gene for complementation | oL3867-promotor-frq-rev | Fw  Rv | AGCGGATAACAATTTCACACAGGAAACAGCACTTCCACCAGCCCACTCCT  TTTCGGAGGATTGCCCTCTGTCGGCTGCATTGTAGGTTCAGGTTATTTAC | 995 |
| *tafrq*-V5His6 | oL3857-oL1586 | Fw  Rv | ATGCAGCCGACAGAGGGCAA  TCAATGGTGATGGTGATGAT | 3143 |
| 3’ UTR with V5 and *bleoR* tail | ol5096-oL5214 | Fw  rv | CGTACCGGTCATCATCACCATCACCATTGATTGTACGGGACATTTCATAT  AATGGAAGTATTAGGGTAGGGTAGGGTAGGATAACAAAAGCTCATTAAAC | 299 |
| *bleoR* | oL5091-oL5090 | Fw  Rv | CAAGCTTGCAAATTAAAGCC  CCTACCCTACCCTACCCTAA | 1307 |
| 3’ flank downstream *tafrq* gene for complementation | oL5213  oL3799 | Fw  Rv | GACGCTCGAAGGCTTTAATTTGCAAGCTTGGCCAGTTTAAGGCTATACTG  GTAACGCCAGGGTTTTCCCAGTCACGACGAAAGTGAGACGAGATAAAGGG | 498 |
