# Supplementary material for: Circadian oscillations in Trichoderma atroviride and the role of core clock components in secondary metabolism, development, and mycoparasitism against the phytopathogen Botrytis cinerea
Source: eLife. 2022 Aug 11;11:e71358. doi: 10.7554/eLife.71358 (PMC9427114; doi:10.7554/eLife.71358)
Supplement: Supplementary file 6. — Fw: direct orientation; Rv: reverse orientation. [file elife-71358-supp6.docx]

**Table S6.** List of primer used for OE::*tafrq* insertion cassette.

 (Fw: direct orientation; Rv: reverse orientation).

| Target DNA | Primer Name | Orientation | Sequence 5’ - 3’ | Size (bp) |
| --- | --- | --- | --- | --- |
| 5’ flank intergenic region *blu17* gene | oL4189  oL3810 | Fw  Rv | GCGGATAACAATTTCACACAGGAAACAGCTAGCGCCTCGTTGCTGAAAT  GACCGGGATCCACTTAACGTTACTGAAATCTATTCGCTACAACGGACAGT | 1500 |
| 3’ flank intergenic region *blu17* gene | oL3813  oL4188 | Fw  Rv | GAGGTGTTTCTTAAGTAGTT  GTAACGCCAGGGTTTTCCCAGTCACGACGAGGACCTATTCTGGAGAGATA | 1500 |
| Complete *tafrq* ORF | oL3857  oL3855 | Fw  Rv | GAAAATTTGCAGGTCGGCATGCCCGTCGGAACCAGGCTAGCCAAACGGCA  ATAACAAATACGTCGTAGGGG | 3053 |
| V5His6 | oL526  oL1586 | Fw  Rv | GGATCTAGAGGGCCCTTCGAA  TCAATGGTGATGGTGATGAT | 93 |
| Actin terminator | oL3856  oL3853 | Fw  Rv | CGTACCGGTCATCATCACCATCACCATTGAAAGCTACGCGAGGTCAAAGA  CAGTCCTGTAAACTACTTAAGAAACACCTCGAATTCCATCTAGCGTTAAAGATACT | 335 |
| Actin promoter | oL3848  oL3854 | Fw  Rv | AAAAATGCTCCTTCAATATCATCTTCTGTCAAAGCGGCCGCTTAGTCGTCAGGAGAGGGAGGC  TTTCGGAGGATTGCCCTCTGTCGGCTGCATTGTGACTGATTATAGGATGA | 1210 |
| *hph* | oL768  oL769 | Fw  Rv | GACAGAAGATGATATTGAAGGAGC  GATTTCAGTAACGTTAAGTGGAT | 1435 |
| Construct amplification | oL83  oL84 | Fw  Rv | GGCAGTGAGCGCAACGCAAT  ATTCAGGCTGCGCAACTGTT | 9126 |
