# Supplementary material for: Circadian oscillations in Trichoderma atroviride and the role of core clock components in secondary metabolism, development, and mycoparasitism against the phytopathogen Botrytis cinerea
Source: eLife. 2022 Aug 11;11:e71358. doi: 10.7554/eLife.71358 (PMC9427114; doi:10.7554/eLife.71358)
Supplement: Supplementary file 7. — Fw: direct orientation; Rv: reverse orientation. [file elife-71358-supp7.docx]

**Table S7.** List of primer used for luciferase translational reporter (TaFRQ^LUC^) insertion cassette.

(Fw: direct orientation; Rv: reverse orientation).

| Target DNA | Primer Name | Orientation | Sequence 5’ - 3’ | Size (bp) |
| --- | --- | --- | --- | --- |
| 5’ flank *tafrq* w/o stop codon | Frq-Luc 5 flank oL3800 | Fw  Rv | GCGGATAACAATTTCACACAGGAAACAGCTACTACAGTGGTGCTCCGT  GCCCTTCTTGATGTTCTTGGCGTCCTCCATGCCACTCCCTGCGGAGCTGGT | 1027 |
| 3’ flank downstream *tafrq* gene | oL3803  oL3804 | Fw  Rv | GACCGGGATCCACTTAACGTTACTGAAATCGCCAGTTTAAGGCTATACTG  GTAACGCCAGGGTTTTCCCAGTCACGACGAAAGTGAGACGAGATAAAGGG | 498 |
| *hph* | oL768  oL769 | Fw  Rv | GACAGAAGATGATATTGAAGGAGC  GATTTCAGTAACGTTAAGTGGAT | 1435 |
| *luc* | oL24  oL25 | Fw  Rv | ATGGAGGACGCCAAGAACAT  TCAGAGCTTGGACTTGCCGC | 1741 |
| *tafrq* 3’UTR | oL3801  oL3802 | Fw  Rv | GCCAAGAAGGGCGGCAAGTCCAAGCTCTGATTGTACGGGACATTTCATATT  AAAAATGCTCCTTCAATATCATCTTCTGTCATAACAAAAGCTCATTAAAC | 299 |
| Construct amplification | oL83  oL84 | Fw  Rv | GGCAGTGAGCGCAACGCAAT  ATTCAGGCTGCGCAACTGTT | 5000 |
