# Supplementary material for: Circadian oscillations in Trichoderma atroviride and the role of core clock components in secondary metabolism, development, and mycoparasitism against the phytopathogen Botrytis cinerea
Source: eLife. 2022 Aug 11;11:e71358. doi: 10.7554/eLife.71358 (PMC9427114; doi:10.7554/eLife.71358)
Supplement: Supplementary file 8. — Fw: direct orientation; Rv: reverse orientation. [file elife-71358-supp8.docx]

**Table S8.** List of primers used for luciferase transcriptional reporter insertion cassette.  (Fw: direct orientation; Rv: reverse orientation).

| Target DNA | Primer Name | | Orientation | | Sequence 5’ - 3’ | | Size (bp) | |  |
| --- | --- | --- | --- | --- | --- | --- | --- | --- | --- |
| 5’ flank intergenic region *blu17* gene | | oL4189  oL3810 | | Fw  Rv | | GCGGATAACAATTTCACACAGGAAACAGCTAGCGCCTCGTTGCTGAAAT  GACCGGGATCCACTTAACGTTACTGAAATCTATTCGCTACAACGGACAGT | | 1500 | |
| 3’ flank intergenic region *blu17* gene | | oL3813  oL4188 | | Fw  Rv | | GAGGTGTTTCTTAAGTAGTT  GTAACGCCAGGGTTTTCCCAGTCACGACGAGGACCTATTCTGGAGAGATA | | 1500 | |
| Putative *sod* promoter | | oL4898  oL4899 | | Fw  Rv | | TAACCTGCACACTGTCCGTTGTAGCGAATGCGGCACCCAGCCTCGGCTTA  GCCCTTCTTGATGTTCTTGGCGTCCTCCATTGTGGCGGTTGGATTTTTAC | | 485 | |
| Putative *con10* promoter | | oL4915  oL4916 | | Fw  Rv | | TAACCTGCACACTGTCCGTTGTAGCGAATGGATGAACATTGATAGTGCAC  GCCCTTCTTGATGTTCTTGGCGTCCTCCATGGTGAAATATGCTTGGTTTG | | 1000 | |
| Putative *ccg9* promoter | | oL4896  oL4897 | | Fw  Rv | | TAACCTGCACACTGTCCGTTGTAGCGAATGAGGAAGCGTACATGTAAATT  GCCCTTCTTGATGTTCTTGGCGTCCTCCATTTTGGAAATCAAGGACTCAA | | 768 | |
| Putative *gpdh* promoter | | oL4894  oL4895 | | Fw  Rv | | TAACCTGCACACTGTCCGTTGTAGCGAATGGTTGGTGCCGGGTCCCAGCC  GCCCTTCTTGATGTTCTTGGCGTCCTCCATGATGGCGGTTTTGTGAACTG | | 1159 | |
| *c-box* from *N. crassa* | | oL3920  oL3921 | | Fw  Rv | | AAAAATGCTCCTTCAATATCATCTTCTGTCCGGAATTATACGATTTAGGT  GCCCTTCTTGATGTTCTTGGCGTCCTCCATATTCTAGTGGAAAGGGGAGG | | 505 | |
| *luc* | | oL24  oL25 | | Fw  Rv | | ATGGAGGACGCCAAGAACAT  TCAGAGCTTGGACTTGCCGC | | 1741 | |
| *hph* | | oL768  oL769 | | Fw  Rv | | GACAGAAGATGATATTGAAGGAGC  GATTTCAGTAACGTTAAGTGGAT | | 1435 | |
