# Supplementary material for: Circadian oscillations in Trichoderma atroviride and the role of core clock components in secondary metabolism, development, and mycoparasitism against the phytopathogen Botrytis cinerea
Source: eLife. 2022 Aug 11;11:e71358. doi: 10.7554/eLife.71358 (PMC9427114; doi:10.7554/eLife.71358)
Supplement: Supplementary file 9. — Fw: direct orientation; Rv: reverse orientation. [file elife-71358-supp9.docx]

**Table S9.** List of primers used for *frq::tafrq^V5^* replacement cassette in *N. crassa* and diagnostic PCRs.

 (Fw: direct orientation; Rv: reverse orientation).

| Target DNA | Primer Name | Orientation | Sequence 5’ - 3’ | Size (bp) |
| --- | --- | --- | --- | --- |
| 5’ flank upstream *ncfrq* (includes native 5’UTR) | oL3670  oL6404 | Fw  Rv | GCGGATAACAATTTCACACAGGAAACAGCGTCAGTACCTCCATCTCACC  TTTCGGAGGATTGCCCTCTGTCGGCTGCATGTTCACCCTATCAGATCTTC | 1382 |
| *tafrq-V5-His6* ORF | oL6405  oL6406 | Fw  Rv | ATGCAGCCGACAGAGGGCAA  AGGCTTACCTTCGAAGGGCCCTCTAGATCCGCCACTCCCTGCGGAGCTGG | 3122 |
| Native 3’UTR *ncfrq* + *bar* resistance cassette + 3’flank  (from pCB05 plasmid) | LF99  oL6378 | Fw  Rv | GGATCTAGAGGGCCCTTCGA  GGTAACGCCAGGGTTTTCCCAGTCACGACGGGTTGCCATTCTGAGTATGG | 2477 |
| *tafrq* | oL3915  oL3916 | Fw  Rv | GAAAATTTGCAGGTCGGCATGCCCGTCGGAACCAGGCTAGCCAAACGGCA  ATAACAAATACGTCGTAGGGG | 1080 |
| *ncfrq* | oL5867  oL5868 | Fw  Rv | AAGATACCGAGGATGGCTCGGATAAGAATGGTCGG  GGCGATGAATTCGGCCGTATCTTCCGATGTTGTCG | 770 |
| 5’ integration of *frq::tafrq^V5^* | AGF133  oL3914 | Fw  Rv | CGCCGGCCCCAGTCTGAATC  TCCGACGGGCATGCCGACCT | 2527 |
