# Supplementary material for: Circadian oscillations in Trichoderma atroviride and the role of core clock components in secondary metabolism, development, and mycoparasitism against the phytopathogen Botrytis cinerea
Source: eLife. 2022 Aug 11;11:e71358. doi: 10.7554/eLife.71358 (PMC9427114; doi:10.7554/eLife.71358)
Supplement: Supplementary file 10. [file elife-71358-supp10.docx]

**Table S10.** RT-qPCR primers used and settings conditions.

| Gene ID | Usual Name | Set of primers | Sequence  5’-3’ | Efficiency | CT frange | Primer Concentration | Mg^2+^ Concentration | Annealing TM (°C) |
| --- | --- | --- | --- | --- | --- | --- | --- | --- |
| TRIATDRAFT_131340 | *tafrq* (total) | oL3750oL3751 | ACTTCATGGTGGTGGTAACG  TAGCCAGCCGATGGATAATG | 94.7 | 14.74/31.5 | 200mnM | 3mM | 60 |
| TRIATDRAFT_301521 | *cytrate lyase* | oL4291oL4292 | AAGATCTTTGGCCCCGAGATG  TTGAACTCCTCCAGGTTGCTG | 91.3 | 7.71/28.98 | 200nM | 3mM | 60 |
| TRIATDRAFT_53190 | *DNApolB* | oL4281oL4282 | GCACTGCGAGGTTATTTGCA  TCTGGAAAGCTCCTTGCCTG | 94.6 | 10.42/27.61 | 200nM | 3mM | 60 |
